# A Case of Spontaneous Osteonecrosis of the Knee with Early and Simultaneous Involvement of the Medial Femoral Condyle and Medial Tibial Plateau

**DOI:** 10.1155/2016/2574975

**Published:** 2016-05-03

**Authors:** Shinya Fujita, Yuji Arai, Kuniaki Honjo, Shuji Nakagawa, Toshikazu Kubo

**Affiliations:** Department of Orthopaedics, Graduate School of Medical Science, Kyoto Prefectural University of Medicine, Kawaramachi-Hirokoji, Kamigyoku, Kyoto 602-8566, Japan

## Abstract

Spontaneous osteonecrosis of the knee (SPONK) usually involves a single condyle, most often the medial femoral condyle (MFC). Involvement of the medial tibial plateau (MTP) is less common, occurring in about 2% of knees with SPONK. Early onset SPONK on the ipsilateral side of the medial compartment is very rare, with, to our knowledge, only four cases reported to date. We describe a very rare case of SPONK with early simultaneous development in the MFC and MTP. Serial plain radiographs and magnetic resonance imaging showed that SPONK in both condyles followed a similar progressive course. The pathological findings in these lesions were similar to those observed in subchondral insufficiency fractures.

## 1. Introduction

Osteonecrosis of the knee can be categorized as primary spontaneous osteonecrosis of the knee (SPONK) [1] or secondary osteonecrosis of the knee associated with a variety of risk factors including use of steroids or alcohol [2, 3]. Secondary osteonecrosis is usually observed in younger patients and involves multiple condyles, whereas SPONK usually develops suddenly in the medial femoral condyle (MFC) of patients aged >55 years [4]. SPONK may also occur in the lateral femoral condyle, medial tibial plateau (MTP), or patella [5]. Involvement of the MTP is observed in only 2% of knees with SPONK [6, 7], whereas early concomitant involvement of the MFC and the adjacent MTP is very rare.

Although the etiology of SPONK remains unclear, it may be due to vascular injury and/or antecedent trauma [8–12], although a recent report suggested that SPONK may result from subchondral insufficiency fractures [10]. Early diagnosis of SPONK is difficult because plain radiographs may be negative, especially if the symptoms are of short duration. Magnetic resonance imaging (MRI) can detect early changes in the subchondral area and assist in the diagnosis of SPONK. This report describes a very rare case of SPONK simultaneously involving the MFC and the MTP and associated with subchondral insufficiency fractures. This condition was diagnosed on MRI soon after symptom onset.

## 2. Case Report

**Figure 1:**
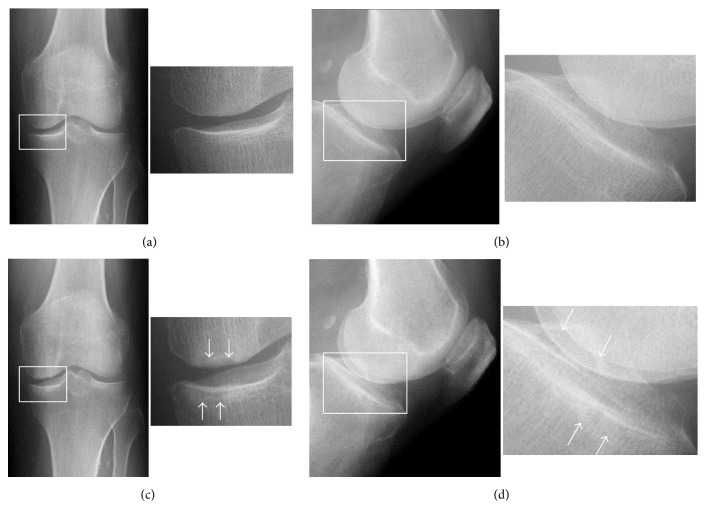
Plain initial (a) frontal and (b) lateral radiographs, showing slight subchondral bone sclerosis on the tibial medial condyle, but no evidence of radiolucent lesions or joint space narrowing. (c) A frontal radiograph and (d) its enlarged view, taken 3 months after the first evaluation, showing a radiolucent oval shadow in the subchondral areas of the weight-bearing portions of both the MFC and MTP, surrounded by a sclerotic halo (arrow).

**Figure 2:**
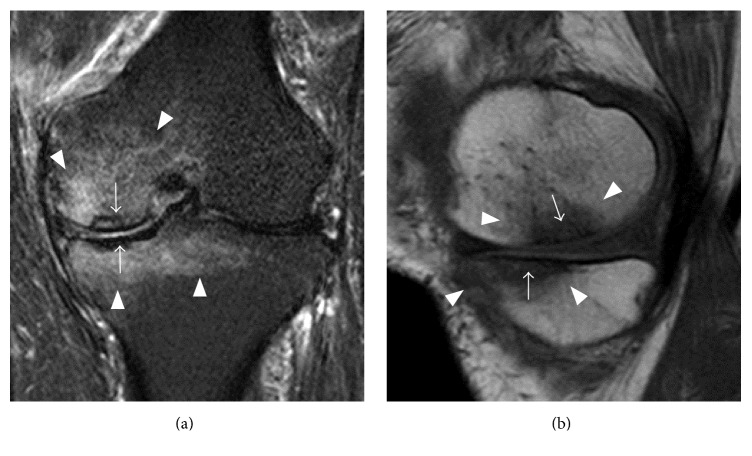
Initial MRI evaluation of our patient. (a) Coronal STIR and (b) sagittal T1 weighted images showing focal SPONK lesions at both the MFC and the MTP (arrow), surrounded by diffuse bone edema (arrowhead). MRI also showed a horizontal tear in the medial meniscus at the posterior horn.

A 72-year-old man presented for evaluation of left knee pain of one-month duration. His previous medical history included coronary artery bypass surgery (CABG) in 2004 after a myocardial infarction. He described a sudden onset of severe pain localized to the medial aspect of his left knee. Severe pain continued for 6 months and did not improve. He had no history of previous trauma, meniscus surgery, steroid treatment, or excessive alcohol use. Physical examination showed tenderness at the medial femoral condyle and medial tibial condyle and slight limitations in the range of motion of the knee. Plain radiographs revealed slight subchondral bone sclerosis on the tibial medial condyle, but the lesion was not radiolucent and there was no evidence of joint space narrowing (Figures 1(a) and 1(b)). Short inversion time inversion recovery (STIR) MRI showed characteristic focal high intensity with band-like low signal intensity portions in the subchondral areas of both the MFC and the MTP, surrounded by diffuse high signal intensity (Figure 2(a)). T1-weighted images showed corresponding focal low intensity lesions (Figure 2(b)), with a horizontal tear present in the medial meniscus at the posterior horn. The patient was diagnosed with SPONK on both sides of the medial compartment. Initial nonsurgical treatment included restricted weight bearing on the affected lower leg.

**Figure 3:**
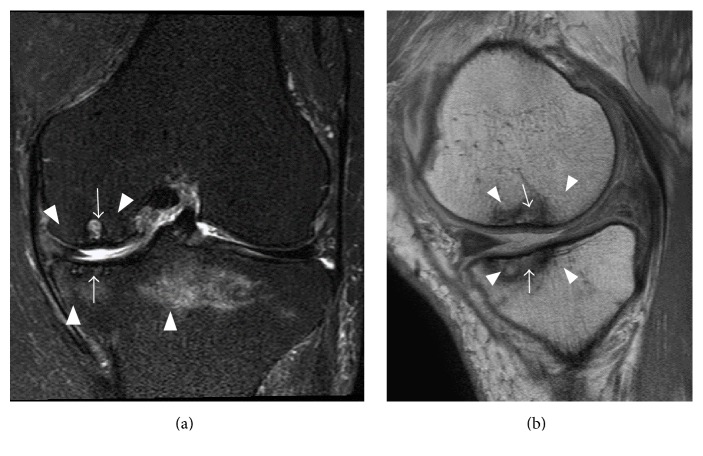
MRI 4 months after first evaluation. (a) Coronal SPIR and (b) sagittal proton density-weighted images showed persistent focal SPONK lesions of both the MFC and the MTP, but the surrounding bone edema had decreased (arrow).

Radiographs three months later showed subchondral radiolucency of the ipsilateral lesions of the medial compartment of the knee. These findings of the MFC and MTP were consistent with Koshino's stage 3 and Carpintero's stage II [6, 12] (Figures 1(c) and 1(d)). Spectral presaturation with inversion recovery (SPIR) MRI 4 months after the initial visit showed band-like low intensity, with a cystic high intensity lesion, in the MFC and a discrete low intensity area, with decreased surrounding high intensity, in the MTP (Figure 3(a)). Proton density-weighted images showed collapse of both the MFC and the MTP at the lesion sites (Figure 3(b)).

**Figure 4:**
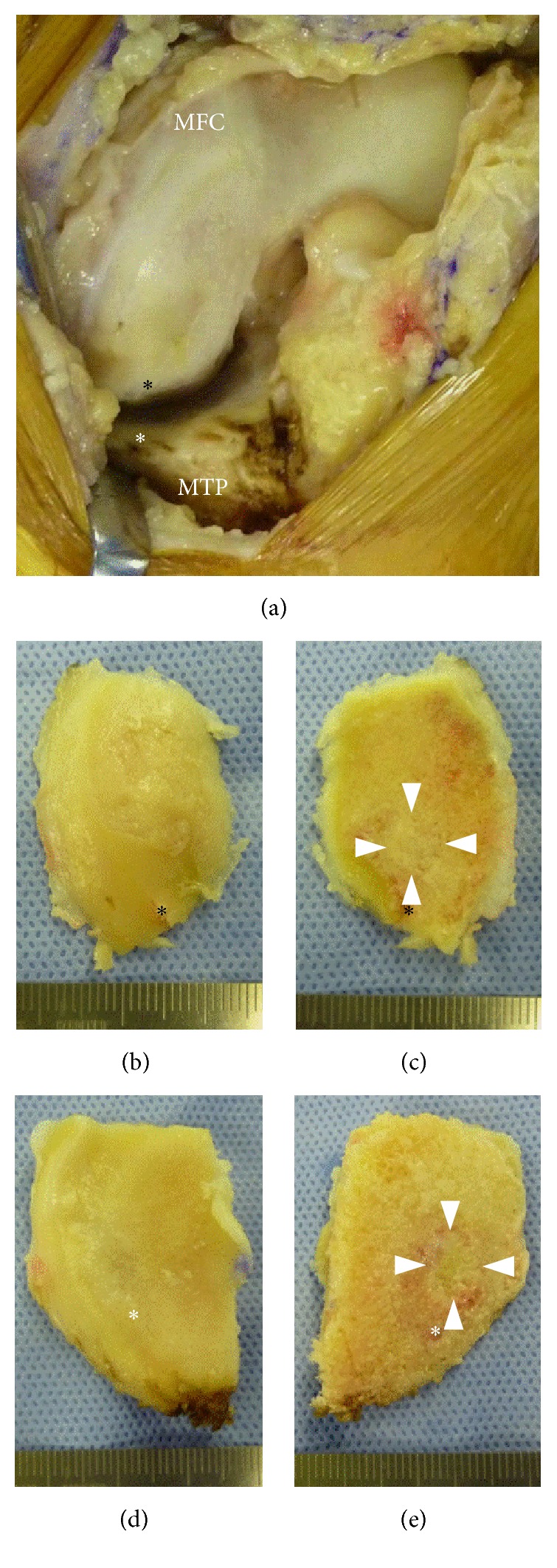
Intraoperative findings. (a) The black asterisk shows the posterior end of SPONK in the MFC, and the white asterisk shows the anterior end of SPONK in the MTP. (b, d) Articular and (c, e) back sides of the (b, d) MFC and (d, e) MTP specimens obtained during UKA. Focal lesions were clearly present in (c) and (e) (arrowhead).

**Figure 5:**
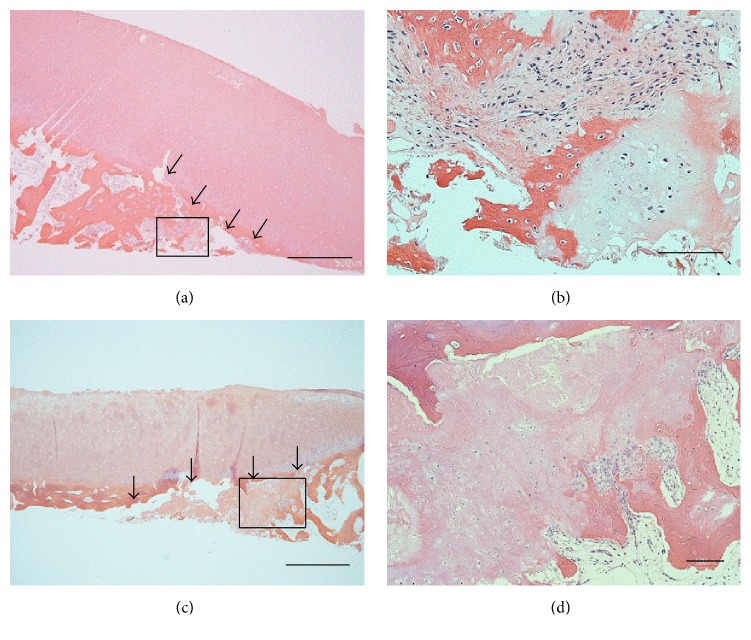
Histological findings (hematoxylin and eosin staining). (a) MFC specimen (original magnification, ×20; bars = 1000 *µ*m), (b) magnification of the square region in (a) (original magnification, ×100; bars = 200 *µ*m). (c) MTP specimen (original magnification, ×20; bars = 1000 *µ*m), and (d) magnification of the square region in (c) (original magnification, ×40; bars = 100 *µ*m). Histological examination of both specimens revealed fracture lines (arrow).

Despite treatment injections of hyaluronan and oral administration of nonsteroidal anti-inflammatory drug, the patient's symptoms did not improve. Because of the persistence of knee pain and the progressive course of his disease, as shown by imaging results, unicompartmental knee arthroplasty (UKA) was performed. UKA was chosen because the prosthesis would be stabilized. Intraoperative examination showed that the articular cartilage at the lesion site in the MFC was smooth but slightly depressed, whereas the cartilage at the lesion site in the MTP had no abrasions and slight fibrillation and was depressed about 1 mm (Figure 4). Parts of the cartilage layers at the affected sites of both the MFC and the MTP obtained during macroscopic UKA had delaminated from the underlying subchondral bone and easily yielded to pressure. Softening of the subchondral bone at the affected site was observed. Osteotomy of the MFC and MTP resulted in complete excision of the lesions. Histological examination of both specimens showed a subchondral crack, fibrous granulation, and osteoid formation, but no evidence of antecedent osteonecrosis (Figure 5). These findings were consistent with those of subchondral fractures. The articular surface of the MFC lesion was smooth, whereas the cartilage of the MTP lesion showed superficial irregularities and degenerative changes. Three years after surgery, this patient has free range of knee motion and no knee pain.

## 3. Discussion

SPONK has been characterized clinically, radiologically, and pathologically. In the natural course of typical SPONK, early stage radiographs may be almost normal, despite the occurrence of intense symptoms (stage 1) [12]. Later, plain films show a typical radiolucent oval shadow in the subchondral area of the weight-bearing portion of the MFC (stage 2), followed by expansion of the shadow and the formation of a surrounding sclerotic halo (stage 3). These structural changes subsequently induce ipsilateral lesions of the subchondral bone and articular cartilage at the MTP, including osteophytes, osteosclerosis, and joint space narrowing (stage 4). Radiographs show the development or progression of joint destruction. If patients present late, the true state of SPONK may be masked and difficult to distinguish from severe osteoarthritis (OA) [13, 14].

MRI provides more extensive information and is both more sensitive and specific in evaluating early SPONK than plain radiographs. In patients with early SPONK, T1 imaging usually shows a focal, semioval low signal intensity, whereas T2 imaging shows subchondral high intensity signals surrounded by band-like low intensity signals [15, 16].

In the present patient, both the MFC and the MTP lesions followed a similar progressive course. SPONK was unremarkable, with normal findings on initial plain radiographs, whereas MRI revealed the characteristic features of these lesions. Focal lesions of both the MFC and the MTP were first observed on plain radiographs 3 months after initial presentation, and the articular surfaces of these lesions were later found to be deformed or damaged. The initial lesion in patients with advanced SPONK displaying simultaneous involvement of the MFC and MTP is difficult to determine [17]. Although cooccurrence of lesions at the MFC and MTP has been reported in 27% to 38% of all patients, most are not early stage lesions at first presentation [6, 17–19]. To our knowledge, only one report found subtle radiographic changes in ipsilateral lesions at the first visit of four patients, suggesting that these lesions were early stage [19]. Our report is unique, in that early imaging findings suggested that SPONK had occurred simultaneously at both sites, with lesions in the MFC and MTP showing similar progression to advanced stage.

The natural course of SPONK of the MFC has been described [20]. Lesion size has been regarded as prognostic of the risk of OA [20]. Patients in whom ≥40% of the joint surface was affected in the AP view have been reported to develop OA [21]. In contrast, the natural course of SPONK of the MTP has been found to range from complete reconstitution to progressive joint degeneration [22]. Extensive collapse of the MTP is rare. Total knee arthroplasty has been reported to be successful in patients with extensive bone collapse, with 97% of these patients continuing to show successful clinical outcomes 9 years after arthroplasty [23]. Our patient had SPONK on both sides of the MFC and the MTP, with the lesions being small and limited to the medial compartment. Unicompartmental knee arthroplasty may be performed if the lesion can be resected.

The precise etiology underlying the initial onset of SPONK has not been clearly determined. Current smokers were reported to be at increased risk for SPONK [24], in agreement with findings showing that many patients with OA smoke an excessive number of cigarettes [25]. Our patient smoked 20 cigarettes per day for 50 years. Smoking may be the cause of simultaneous involvement of the MFC and MTP.

Histologically, SPONK has been found to differ from avascular necrosis in the femoral head. Rather, histological findings in early stages of SPONK were shown to be identical to those of subchondral fractures, with no evidence of antecedent osteonecrosis, and focal areas of necrosis distal to the fracture line in advanced stages of SPONK have been considered a secondary condition after subchondral fractures [10]. The histological features of advanced lesions were reported to closely resemble those of delayed union or nonunion, with the resulting formation of cartilage and fibrous tissue [26]. Impaired healing after a subchondral fracture resulted in an unstable detached fragment distal to the fracture and loss of blood supply, resulting in osteonecrosis. These histological features are consistent with a history of acute onset and a radiological variant in advanced stages of SPONK, suggesting that subchondral insufficiency fracture is the most likely underlying cause. Histological findings in our patient included a subchondral crack and bone repair reactions, such as fibrous granulation tissue and osteoid formation without osteonecrosis. Therefore, SPONK in this patient was likely due to a subchondral insufficiency fracture at the MFC and MTP.

Medial meniscal tears have been observed in 50% to 78% patients with SPONK [16, 27, 28]. Mechanical environmental changes in the medial meniscus can increase contact stresses across the joint and produce focal subchondral overload, which may predispose to the development of osteonecrosis [28–31]. Meniscal damage may play a role in the concomitant occurrence of osteonecrosis of the MFC and MTP [32]. Meniscal root injuries have been reported in 80% of patients with SPONK and meniscal tears in 67%, indicating that loss of hoop stress in meniscal root injuries altered meniscal function, increasing pressure on the subchondral bone [33]. Our patient showed a horizontal tear in the medial meniscus at the posterior horn, but no root injury. However, the loss of meniscal function due to the horizontal tear may have been responsible, at least in part, for the concurrent development of SPONK at the MFC and MTP.

## 4. Conclusion

This report describes a patient with SPONK concomitant with ipsilateral lesions of the medial compartment from early onset. The major findings in the present patient were subchondral insufficiency fractures in the MFC and MTP.
